# Supplementary material for: FERN – a Java framework for stochastic simulation and evaluation of reaction networks
Source: BMC Bioinformatics. 2008 Aug 29;9:356. doi: 10.1186/1471-2105-9-356 (PMC2553347; doi:10.1186/1471-2105-9-356)
Supplement: Additional file 1 — FERN distribution, Version 1.3. This archive contains the FERN source code and binaries as well as documentation and example models in FernML and SBML. [file 1471-2105-9-356-S1.zip › fern/doc/javadoc/fern/simulation/controller/package-use.html]

Uses of Package fern.simulation.controller


---


|  |  |  |
| --- | --- | --- |
| **Overview** \| **Package** \| Class \| **Use** \| **Tree** \| **Deprecated** \| **Index** \| **Help** | |  |
| PREV   NEXT | **FRAMES**    **NO FRAMES**     **All Classes** |  |


---


## **Uses of Package fern.simulation.controller**

| Packages that use fern.simulation.controller | |
| --- | --- |
| **fern.benchmark** | Provides classes for benchmarking simulations (time benchmarks, histogram distance calculation). |
| **fern.simulation** | Provides algorithms for simulating reaction network as well as methods for observer certain aspects of a simulation. |
| **fern.simulation.algorithm** | Provides algorithms for stochastic simultion of reaction networks. |
| **fern.simulation.controller** | Provides classes to control the simulations. |
| **fern.simulation.observer** | Provides classes to observe certain aspects of simulations. |

Classes in fern.simulation.controller used by fern.benchmark

****SimulationController****
  
          `Simulator`s are controlled by implementing classes.

Classes in fern.simulation.controller used by fern.simulation

****SimulationController****
  
          `Simulator`s are controlled by implementing classes.

Classes in fern.simulation.controller used by fern.simulation.algorithm

****SimulationController****
  
          `Simulator`s are controlled by implementing classes.

Classes in fern.simulation.controller used by fern.simulation.controller

****SimulationController****
  
          `Simulator`s are controlled by implementing classes.

Classes in fern.simulation.controller used by fern.simulation.observer

****SimulationController****
  
          `Simulator`s are controlled by implementing classes.

---


|  |  |  |
| --- | --- | --- |
| **Overview** \| **Package** \| Class \| **Use** \| **Tree** \| **Deprecated** \| **Index** \| **Help** | |  |
| PREV   NEXT | **FRAMES**    **NO FRAMES**     **All Classes** |  |


---
